# Reinvesting the cellular properties of human amniotic epithelial cells and their therapeutic innovations

**DOI:** 10.3389/fimmu.2024.1466529

**Published:** 2024-10-15

**Authors:** Jing Yang, Yuefeng Lu, Jinping Zhao, Yi Luo, Wangping Hao, Wencheng Zhang, Zhiying He

**Affiliations:** ^1^ Institute for Regenerative Medicine, Medical Innovation Center and State Key Laboratory of Cardiology, Shanghai East Hospital, School of Life Sciences and Technology, Tongji University, Shanghai, China; ^2^ Institute of Cellular Drug Development, Shanghai iCELL Biotechnology Co., Ltd, Shanghai, China; ^3^ Shanghai Engineering Research Center of Stem Cells Translational Medicine, Shanghai, China; ^4^ Shanghai Institute of Stem Cell Research and Clinical Translation, Shanghai, China

**Keywords:** human amniotic epithelial cells (hAECs), cell therapy, epithelial-mesenchymal plasticity (EMP), cell expansion, clinical trials, therapeutic mechanisms

## Abstract

Human amniotic epithelial cells (hAECs) have shown promising therapeutic effects in numerous studies on various diseases due to their properties such as low immunogenicity, immunomodulation, paracrine effect, and no teratoma formation *in vivo*. Nevertheless, there are still many problems in archiving the large-scale clinical application of hAECs, ranging from the vague definition of cell properties to the lack of clarification of the motion of actions in cell therapies, additionally, to the gap between cell quantities with limited proliferation capacity. This review provides a detailed overview of hAECs in the aspects of the lineage development of amniotic epithelial cell, cell characteristics and functional roles, *ex vivo* cell cultivation and expansion systems, as well as their current status and limitations in clinical applications. This review also discusses the advantages, limitations and feasibility of hAECs, and anticipates their prospects as cell therapy products, with the aim of further promoting their clinical applications.

## 1 Introduction

Human amniotic epithelial cells (hAECs) are derived from the amniotic ectoderm which differentiates from the epiblast around day 8 after fertilization. The cell morphology and transcriptome of amniotic ectoderm are distinctly different from epiblast. As the differentiation and expansion proceed, amniotic ectoderm gradually gets apart from epiblast, forming amniotic cavity filled up with amniotic fluids, and this structure is called amniotic sac. Amniogenesis occurs prior to the formation of primitive streak, the hallmark of the initiation of gastrulation, making amniotic ectoderm cells one of the primordial extraembryonic cells. hAECs are tightly packed as a monolayer, constituting the innermost layer of amnion, directly in contact with amniotic fluid (1, 2). It has been reported that pluripotency markers such as TRA1-60, TRA1-81, SSEA3, and SSEA4 are expressed in hAECs isolated from early gestational amnion, then are gradually lost over the pregnancy period (3). Although many clinical studies have shown that hAECs are of promising therapeutic potential in various diseases, the specific cell biological features and mechanisms for the treatments still remain elusive. In addition, it has been shown that hAECs have limited proliferation capability due to the scarce telomerase activity, impeding their development as a cellular product in the large-scale clinical applications and their further industrialization. To address these problems, it is critical to have a comprehensive understanding of hAECs, including cellular properties, the intrinsic proliferative capacity, and the therapeutic potentials.

## 2 Lineage maturation of human amniotic epithelial cells

**Figure 1:**
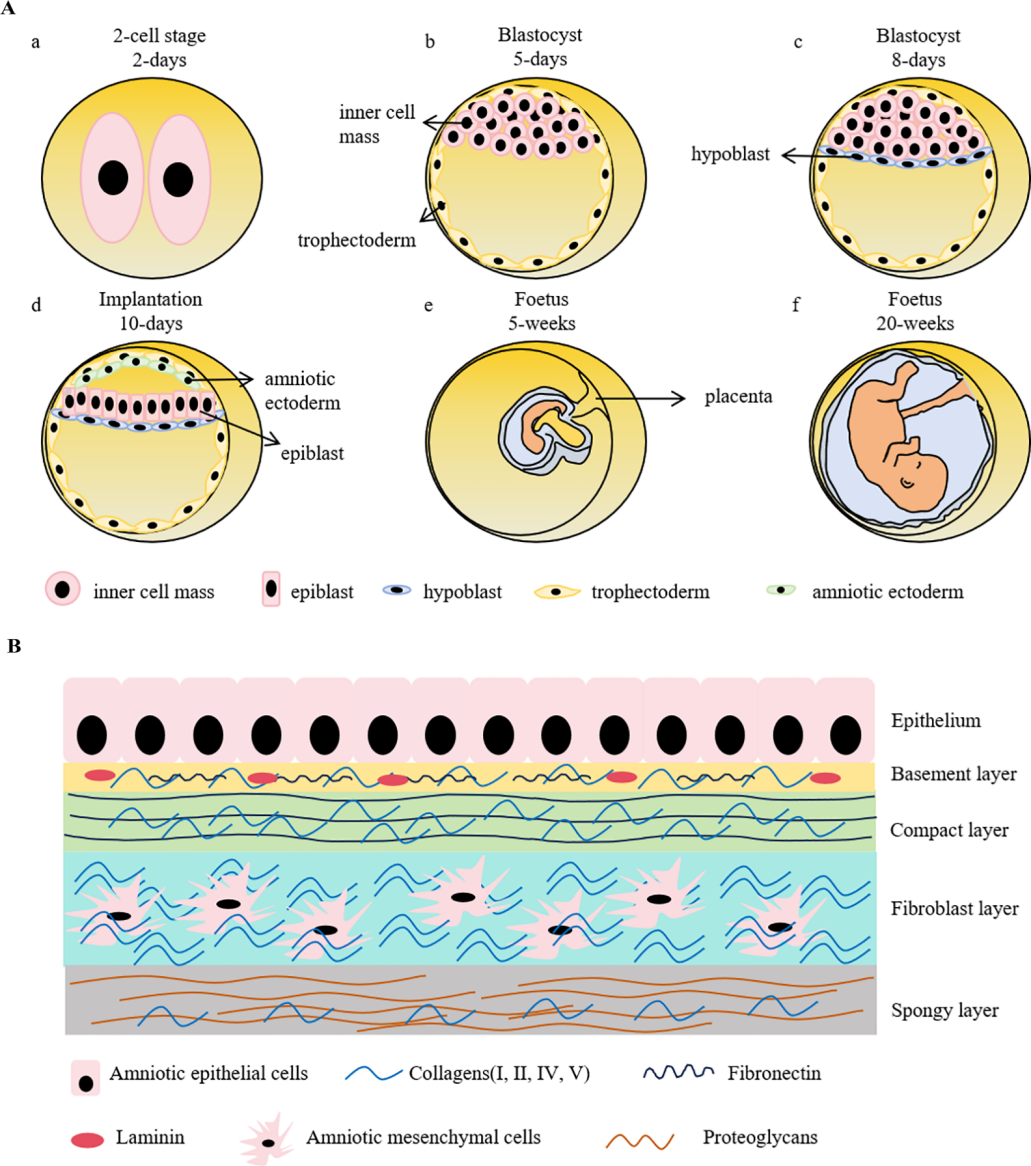
Schematic representation of early embryonic development and the structure of human amniotic membrane. **(A)**. A zygote goes through rapid divisions. At the morula stage, the cells exhibit differential division rates, resulting in the formation of inner cell mass (ICM) and trophectoderm. ICM further differentiates into epiblast and hypoblast, forming the bilaminar disk prior to the embryo implantation. Amniotic sac is instantly developed during peri-implantation, which precedes the formation of primitive streak, the hallmark of gastrulation. Hypoblast develops into yolk sac to provide nutrients for the early embryo development before the maturation of placenta. Trophectoderm, on the other side, forms trophoblast and then eventually develops into placenta. a-d shows the stages of early embryonic development. **(B)**. Human amniotic membrane is composed of 5 layers: 1) an epithelial monolayer, 2) a basement membrane layer, 3) a compact layer, 4) a fibroblast layer and 5) a spongy layer. hAECs are arranged on the basement membrane which is mostly made up by collagen (type III, IV, V), fibronectin and laminin. The compact layer is the main fibrous skeleton containing collagen (type I, III, V, VI) and fibronectin. Human amniotic mesenchymal cells locate in the fibroblast layer consisting of collagen (type I, III, IV), fibronectin, laminin and nidogen. The spongy layer as the intermediates between amnion and chorion are mainly comprised collagen (type I, III, IV) and proteoglycans.

Amniotic epithelial cells are derived from epiblast around day 8 post fertilization. During the early embryogenesis, a zygote is a totipotent cell having the potentials to develop into both embryonic and extra-embryonic tissues. Once it forms, it rapidly undergoes successive divisions including the 2-cell, 4-cell, 8-cell, 16-cell (morula), and blastocyst stages. At the morula stage, the cells exhibit differential division rates that faster-dividing cells forming a non-polarized inner cell mass (ICM) in the interior of embryo while the slow-dividing cells tightly aligning in the exterior to encompass the ICM and eventually differentiating into trophectoderm (TE), forming the blastocyst. As the blastocoel expands, the ICM predominantly aggregates on one side of the embryo, establishing an overall polarized embryo (4, 5). The ICM further differentiates into epiblast and hypoblast (primitive endoderm), forming the bilaminar disk. Amniotic ectoderm, is believed as the primordial amniotic epithelial cells, emerges during peri-implantation from the differentiation of epiblast. Subsequently, the primitive streak will arise from the derivation of the non-amniotic origin epiblast, which represents the initiation of the three embryonic germ-layers and organogenesis (**Figure 1A**).

The amnion, a crucial extraembryonic tissue, is an important milestone in animals’ evolutionary transitions from aquatic to terrestrial environment. Amnion provides mechanical protection to the fetus and secretes cytokines and hormones, contributing to the embryo development. Although the underlying mechanisms of amniogenesis and amniotic epithelial lineage development are not fully understood yet, the specifications of amniogenesis have been reported with two different patterns: folding and cavitation (6). In species such as bats, monkeys, and higher primates, the amnion emerges by delamination from pluripotent epiblast around the peri-implantation, followed by epithelialization and cavitation, forming the amniotic sac (7, 8). In contrast, in mice, rabbits, Pteropodid bats, dogs, pigs, cows, and lower primates, the amnion is formed by the folding of embryonic tissues during or shortly after gastrulation, whereby the amniotic folds extend and merge to form a closed sac (9–11). It is noteworthy that despite these different amniogenesis patterns, the anatomical structures and functions of amnion are similar across various species. Because of the ethical concerns, the investigations of human amniotic epithelial cells lineage development were restricted in the stage of formation of amniotic ectoderm. The entire process remains unclear; therefore, the substitute models using pluripotent stem cells *in vitro* have been established to learn its lineage development and to comprehensively understand the amniotic epithelial cells biological features and therapeutic potentials as a promising cellular drug candidate.

Previous studies have employed single-cell transcriptome sequencing (scRNA-seq) to analyze three stages of primate embryonic development, including the cultured human pre-gastrulation embryos, *in vitro* cultured Cynomolgus monkey gastrulating embryos, and a human gastrulating embryo implanted *in utero*. The integrated scRNA-seq analysis revealed that amniogenesis occurred in two distinct waves of epiblast differentiation during early embryonic development. The two waves occur independently and develop in different routes, amniotic epithelial cells-early (AME-E) and amniotic epithelial cells-late (AME-L), respectively. AME-E follows a trophectoderm-like route and the amniotic cavity is formed during the early wave, whereas AME-L follows a nonneural ectoderm-like transcriptional program (9). Similar results were also observed in the differentiation of human pluripotent stem cells (hPSCs) from different states. The naïve and primed hPSCs could model the two waves of amniogenesis (9).

Due to the “14-day rule”, referring to the International Society for Stem Cell Research (ISSCR) recommendation that human embryos created by *in vitro* fertilization, either frozen or unfrozen, cannot survive outside the body beyond the 14th day after fertilization without embryo transfer, and technological constraints, the studies of embryonic development remain quite limited, as well as a similarly scant comprehension to amniotic development. The amnion is composed of epithelium, basement membrane, the compact stromal layer, fibroblast layer, and the intermediate spongy layer (**Figure 1B**). The amniotic epithelial layer localizes at the innermost of amnion and directly contacts with the amniotic fluid. The protein secreted by amniotic epithelial cells, such as glycoproteins and collagens, constitute the underlying basement membrane (12). Amniotic epithelial cells and amniotic mesenchymal cells primarily reside in the epithelium and the fibroblast layer, respectively (13). These two cell types have distinct origins. Amniotic epithelial cells are derived from amniotic ectoderm (14), in contrast, amniotic mesenchymal cells originate from extra-embryonic mesoderm, which is developed posterior to amniotic epithelial cells (15). As the two major cell types derived from the amnion membrane, both cells have the advantages of low immunogenicity, no tumorigenicity, and limited ethical considerations. The characterized markers of hAECs and human amniotic mesenchymal stem cells (hAMSCs) are listed in **Table 1**. It has been demonstrated that the epithelial markers, such as cytokeratins, E-cadherins, and CD9, are highly expressed in hAECs but not in hAMSCs. Multiple mesenchymal markers like CD90, CD29, and CD105 are also expressed in hAECs, indicating the spontaneous occurrence of epithelial-mesenchymal transition (EMT) during the cell cultivation *in vitro*. Both of them low express HLA-A, -B, -C, and are negative in the detection of hematopoietic stem cell markers, representing their low immunogenic potential. Although some pluripotency markers (SSEA4, OCT4, TRA1-60, and REX1) have been detected in hAECs and hAMSCs, the expression levels are extremely low compared with that of induced pluripotent stem cells (iPSCs).

**Table 1:** Comparative analysis of the properties of hAECs and hAMSCs.

Cell Type	Epithelial Cell Markers	Mesenchymal Stem Cell Markers	Pluripotent Stem Cell Markers	Hematopoietic Stem Cell Markers	MHC and Co-Stimulatory Molecules		References
	Positive	Positive	Positive	Negative	Positive	Negative	
hAECs	Cytokeratin, E-cadherin	CD29, CD166, CD90	OCT4, NANOG, SSEA4, TRA1-60, SOX2, REX1	CD34, CD45, CD31	HLA-ABC	HLA-DR, HLA-DQ	Yang et al. (39)
	CK19	CD29, CD44, CD73, CD90, CD105	SSEA4, OCT4, SOX2	CD31, CD34, CD45, CD49d	N/A	HLA-DR	Wu et al. (125)
	CK7, E-cadherin	CD29, CD73, CD105	OCT4, NANOG, SSEA4	CD34, CD45	HLA-ABC	HLA-DR	Liu et al. (126)
	E-cadherin	N/A	OCT4, SOX2, NANOG, KLF4, SSEA3, SSEA4, TRA1-60, REX1	N/A	N/A	N/A	Castro et al. (127)
	E-cadherin, CK7, EpCAM	Vimentin, CD44, CD90, CD105, CD146, CD29, CD49f	N/A	CD31, CD45	HLA-ABC, CD40	HLA-DP-DQ-DR, CD80, CD86	Pratama et al. (128)
	Cytokeratin	N/A	SOX2, SSEA3, SSEA4, TRA1-60, OCT4, NANOG	CD34	N/A	N/A	Evron et al. (129)
	CD9	CD29, CD104, CD105, CD44, CD90, CD10, CD49f	SSEA3, SSEA4, TRA1-60, TRA1-81, OCT4, NANOG	CD34, CD45	HLA-ABC, CD40	HLA-DR, CD80, CD86, CD40l	Banas et al. (130)
	N/A	CD90	SSEA3, SSEA4, TRA1-60, TRA1-81, REX1	CD34	N/A	N/A	Miki et al. (26)
	CK19, E-cadherin	CD29, CD44, CD90	OCT4, SOX2, SSEA4	CD34, CD45	N/A	N/A	Wu et al. (131)
	E-cadherin, CD9, CD24	CD29, CD49f	ABCG2, SSEA3, SSEA4, TRA1-60, TRA1-81, OCT4, NANOG	CD34, CD133	N/A	N/A	Miki et al. (24)
	N/A	CD90, CD44, CD73, CD166, CD105, CD29, STRO-1	SSEA4	CD34, CD45	N/A	N/A	Dia-Prado and Sugiura et al. (132, 133)
hAMSCs	N/A	CD29, CD44, CD49d, CD73, CD90, CD105	SSEA4, OCT4, SOX2	CD31, CD34, CD45	N/A	HLA-DR	Wu et al. (125)
	N/A	CD29, CD73, CD90, CD105	OCT4, NANOG, SSEA4	CD34, CD45,	HLA-ABC	HLA-DR, CD80, CD86, CD40	Liu et al. (134)
	N/A	CD29, CD73, CD90, CD105	OCT4, NANOG, SSEA4	CD34, CD45, CD133	HLA-ABC	HLA-DR, CD80, CD86, CD40	Li et al. (135)
	N/A	CD44, CD90, CD105, CD146	OCT3/4, REX1	CD45, CD34	HLA-ABC	HLA-DR	Bacenková et al. (136)
	N/A	CD90, CD44, CD73, CD166, CD105, CD29, CD271, STRO-1	SSEA4	CD34, CD45	N/A	HLA-DR	Diaz-Prado et al. (132)
	N/A	CD90, CD44, CD73, CD166, CD105, CD29, CD271, STRO-1	SSEA4	CD34, CD45	N/A	HLA-DR	Sugiura et al. (133)
	N/A	CD29, CD105, CD73, CD90, CD13, CD44, CD166, STRO-1	OCT3/4, SSEA4, SOX2, NANOG, REX1	CD34, CD45, CD31	HLA-A, HLA-DQB1	CD80, CD86, CD40	Mihu et al. (137)
	N/A	CD44, CD73, CD90, CD105, Vimentin	OCT3/4, c-Myc, SOX2, NANOG, SSEA3, SSEA4	CD34, CD45	N/A	HLA-DR	Nogami et al. (138)

Pluripotent stem cells, including embryonic stem cells (ESCs) and iPSCs, have been often employed in the studies of early embryonic development because of their differentiation potentials to the three-germ layers. It has been demonstrated that PSCs possess of the formative naive-to- primed transition *in vitro*. As widely recognized, naive PSCs are akin to the pre-implantation epiblast, having the differentiation potentials for both embryonic and some extra-embryonic tissues, while primed PSCs are more like the post-implantation epiblast and differentially develop into the embryonic tissues (16). Fu and his team established the first 3D model of amniotic sac embryoid using microfluidic devices and human PSCs, known as microfluidic platform for analysis of single embryos (μPASE), to explore the early development of amniotic sac, and found that the activation of BMP-SMAD signaling enabled the self-organization of hPSCs into amniotic sacs (17).

In another study, *Wu* et al. likewise induced the organization of hPSCs into structures resembling to the early embryos, termed Blastoids, which expressed the amniotic genes, exhibiting the morphological similarities to human blastocyst. Unlike μPASE, which collapsed within 48h, Blastoids were able to be maintained up to 4 days *in vitro* (18). Same results were also obtained using iPSCs, named as iBlastoids (19). On the basis of the previous research, Qin and his associates upgraded the microfluidics amniotic sac platform and exerted a perfuse-able microfluidic device to fabricate an advanced 3D amnion microchip. The constant-rate perfusion of culture medium prolonged the presence and integrity of the amniotic sac-like structure to 20 days. It offers a great strategy and model to investigate human amniotic development in mid-gestation (20).

## 3 Characterization and expansion of hAECs

Although the therapeutic effects of hAECs have been evidenced in many clinical studies, their applications are restricted by the current *in vitro* culture expansion systems. The yield and biological characteristics of primary hAECs are affected by various factors like the physical condition of parturients, placental size and quality, origins from different amnion regions, gestation stages, isolation and preservation procedures (3, 21, 22). The average quantity of hAECs isolated from an amnion is approximately 1 × 10^8^, whereas a single treatment typically requires the amount in the range of 0.5~1×10^8^, leading to the homogenous cells might not be sufficient for multiple treatments in one patient. Therefore, the requirement to obtain adequate hAECs complying with Good Clinical Laboratory Practice (GCLP) standards has become an absolute challenge. Developing a system that is sufficient to expand hAECs *in vitro* requires understanding the properties of hAECs, as well as their proliferation mechanisms. However, regardless of the extensive clinical studies of hAECs in various diseases, the lack of typical markers and standard production process for the quality control of hAECs still are the most urgent issues to be addressed.

### 3.1 Cellular markers of hAECs

Unlike the other parts of the placenta, hAECs lineage development occurs prior to the formation of primitive streak. Therefore, it is reasonable to speculate that a certain proportion of hAECs may retain the biological properties of pre-gastrula embryonic cells. Indeed, recent studies have shown that the pluripotency factors NANOG, OCT4, and SOX2 are not completely silenced in hAECs, as their promoters are only partially methylated. hAECs exhibit similar epigenetic profiles compared to hiPSCs, and their post-transcriptional expression is regulated by specific miRNAs (23). Although these stem cell markers are lost over time, a few hAECs may still partially retain them in the full-term amnion, and the expression of these pluripotency markers varies at different cell passages and different culture conditions (24–26). Other than the pluripotency markers, hAECs also low express classical HLA class I molecules (-A, -B, and -C) and non-classical HLA-I molecules (-E, -F, and -G) (27–29). Co-cultivation of hAECs and allogeneic peripheral blood mononuclear cells (PBMC) or T lymphocytes did not stimulate T lymphocytes proliferation. Instead, they reduce the proliferation and activation of T and B lymphocytes and inhibit the functions of active NK cells and T lymphocytes, thus exerting systemic immune regulation (30, 31). Moreover, transplantation of hAECs into human and mice via either the direct injection or intravenous infusion has not resulted into significant occurrences of immune rejections (32, 33), suggesting that hAECs have the low immunogenic property and eminent immunomodulatory effect. Furthermore, hAECs also secrete a variety of soluble immune regulatory factors, such as macrophage migration inhibitory factor (MIF), Transforming growth factor-β (TGF-β), interleukin (IL)-10, prostaglandin E2 (PGE2) and hepatocyte growth factor (HGF), inhibiting the chemotactic migration activities of neutrophils and macrophages (30, 31).

Other studies are also striving to use scRNA-seq and *in vitro* models of embryonic development to discover more markers that can specifically characterize hAECs. By integrating existing sequencing data, including transcriptome and proteome sequencing, and combining them with the studies on amniotic cell phenotypic properties to establish a specific database for amnion-derived cells, these works would facilitate the understandings of hAECs and provide more specific markers when preparing cells for their therapeutic application in disease treatments.

### 3.2 *In vitro* culture systems for hAECs

Ever since the first report of the isolation and cultivation of hAECs by *C. A. Akle* (34), considerable effort has been invested in the area of hAECs isolation and *in vitro* expansion culture systems. The quantity, quality, and biological properties of hAECs are technically challenged by various factors, such as parturient individual heterogeneity (35), size and mass of placenta (36), regional area of placenta (37), and gestational age (21). The effects of these parameters on cell phenotype and marker expression profiles have been discussed in detail (38). To ensure sterility and quality, the placenta has to be obtained from C-section, HBV, HCV, HIV infection and pre-diagnosed genetic abnormalities are excluded. Furthermore, it is crucial to scrape off chorion and the residual blood from amniotic membrane prior to the tissue digestion steps. With a view to the clinical applications, an optimized culture system using xeno-free culture media has been studied and adapted to medical-applied bioproduction of hAECs (39).

However, there is no consolidated protocol adopted in diverse laboratories till now, and the difference ranges from the basal medium, nutrient supplements to cell density and culture conditions (2, 26). On the basis of the numerous studies focusing on refining the *in vitro* cultivation for hAECs, a comprehensive analysis of the outcomes in these studies reveals common issues, including the low cell-matrix adherence activity of primary hAECs, rapid aging, transformation of cells via EMT, and a relatively low cell proliferation coefficient (1, 40). Given the needs of hAECs in the clinical transitional research and applications, an optimized strategy for hAECs large-scale expansion is urgently needed. Although several commercialized products of hAECs have been marketed (**Table 2**), their methodological issues, like exogenous serum, cryopreservation conditions, and the unknown cultivation procedures, have greatly confined their medical translations.

**Table 2:** Currently available commercial products of hAECs.

Product	Brand	Cat.	Info.	Cultivation
HAEpiC	Innoprot	P10957	· HAEpiC were cryopreserved at passage one and delivered in frozen.	Collagen type I-coated vessel.
HAEpiC	ScienCell	7110	· HAEpiC were cryopreserved at passage one and delivered in frozen.	Poly-L-lysine-coated culture vessel (2 μg/cm2. Epithelial Cell Medium (EpiCM, Cat. #4101): EpiCM consists of 500 ml of basal medium, 10 ml of fetal bovine serum (FBS, Cat. No. 0010), 5 ml of epithelial cell growth supplement (EpiCGS, Cat. No. 4152), and 5 ml of Antibiotic Solution (P/S, Cat. No. 0503).
Human Amniotic Epithelial Stem Cells	BIO TREND	HAEC-100	· Human Amniotic Epithelial (HAE) Cells were isolated from the surface layer of the amniotic membrane of fresh placentas.	HyClone media and supplements:cat. SV30103.01.
Human amnion–derived multipotent progenitor (AMP)	Noveome Biotherapeutics, Inc.	N/A	· A novel, cultured cell population derived from AECs, termed human amnion–derived multipotent progenitor (AMP) cells, secrete numerous cytokines and growth factors that enhance tissue regeneration and reduce inflammation. This AMP cell secretome, termed ST266.	N/A
Human Placental Epithelial Cells (HPlEpC)	Creative bioarray	PCELL-0123	· Human Placental Epithelial Cells (HPlEpC) were derived from the inner surface of amniotic membrane and have physiology related to fetal development and neurogenesis. · 500,000 HPlEpC (primary culture) frozen in Basal Medium w/10% FBS, 10% DMSO	Cryovial frozen HPlEpC (230-05), Growth Medium (215-500), Subcltr Rgnt Kit (090K)

To improve hAECs *in vitro* expansion efficiency and acquire abundant cells for clinical therapies, a few innovative approaches have been employed, such as 3D cultivation, cell reprogramming, iPSCs differentiation, and gene editing (41–43). In recent research, multiple biomimetic microcarriers have been constructed and applied to hAECs expansion cultivation *in vitro*. The proliferative capacity of hAECs was better maintained and the amplification was significantly improved. Moreover, the intrinsic mechanisms in the regulation of cell proliferation in hAECs were explored in depth via transcriptome sequencing. The critical pathways involved in hAECs proliferation were then manipulated to achieve cell reprogramming. On the other side, utilizing iPSCs to differentiate into hAECs has also been investigated.

### 3.3 The proliferative mechanisms of hAECs

The primary hAECs isolated from termed-pregnancy amnion are known of lacking the telomerase activity. Typically, telomerase activity is exhibited in stem cells, reproductive cells, and tumor cells. Telomeres and telomerase activity are often implied to cell proliferative capacity. Interestingly, although hAECs do not express TERT, a critical catalytic subunit of telomerase (39, 44), and cannot undergo unlimited proliferation, they are involved in regulating the telomerase activity of human corneal endothelial cells through Wnt/β-catenin pathway, promoting their proliferation (45). Besides, hAECs have relative long-length telomeres compared to bone marrow-derived mesenchymal stem cells (BM-MSCs), but it is less than human embryonic stem cells (hESCs) (46). Exogenously overexpressing TERT would enhance hAECs proliferative capacity but was not enough for the unlimited growth. Down-regulating the expression of p16INK4a and p53, along with activating telomerase, is necessary to establish an immortalized hAECs cell line (42). These indicated that hAECs might achieve cell proliferation via a telomerase-independent mechanism.

The studies revealed that during the limited expansion *in vitro*, hAECs expressed both epithelial and mesenchymal markers, and were considered in pEMT (Partial epithelial mesenchymal transition, pEMT) state (39, 47), suggesting that hAECs might achieve the proliferative capacity via regulating their EMT states (48). EMT refers to the biological process where epithelial cells transform into cells with mesenchymal phenotypic characteristics through a specific program. While pEMT, or EMP (Epithelial mesenchymal plasticity) refers to the intermediate state during EMT process (49, 50). It manifests as cells partially owning both epithelial and mesenchymal characteristics. Recent studies in the field of cancer have found that cells in pEMT, rather than cEMT (complete EMT), are more closely related to cancer stem cells (51). Similarly, the stem cell properties of trophoblast cells also have been reported to be associated with pEMT (52, 53).

**Figure 2:**
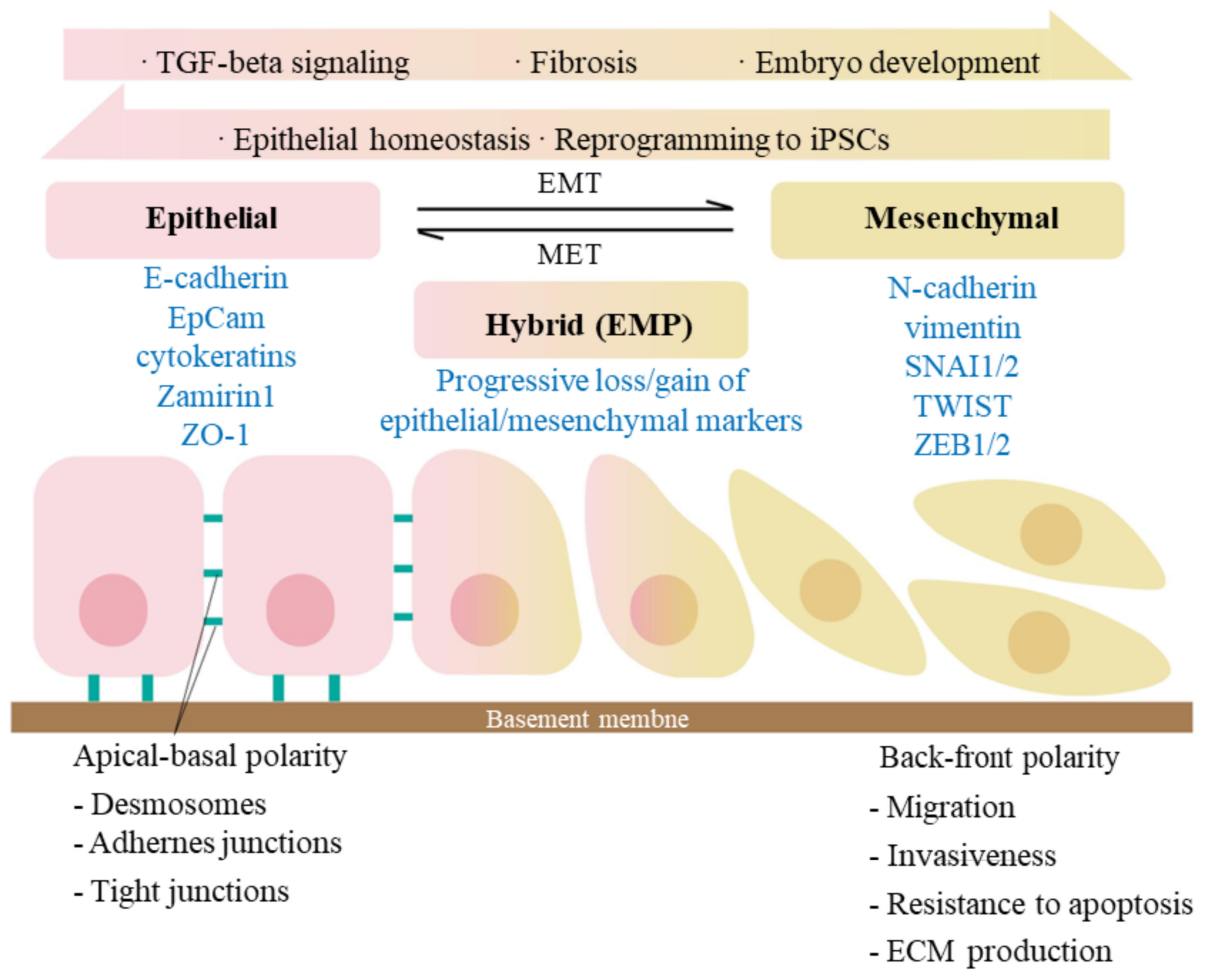
Signaling pathways involved in epithelial-mesenchymal transition (EMT). EMT is a biological process that allows an epithelial cell to undergo phenotypic and biochemical transitions that enable it to present a mesenchymal cell phenotype, losing the interaction with basement membrane while gaining the characteristics like migratory capacity and invasiveness. MET is, on the other side, an exact reversed process. The epithelial and mesenchymal cell markers commonly used are listed. Co-expression of the two sets of distinct markers during EMT indicates an intermediate/hybrid state, termed as EMP. Recent studies have shown that during embryogenesis and epithelia homeostasis, certain epithelial cells appear to be plastic and thus able to move back and forth between epithelial and mesenchymal states via the processes of EMT and MET. The activation of TGF-β signaling has been proved to induce the initiation of EMT.

The typical features of EMT are the reduced expression of cell adhesion molecules (such as E-cadherin), transformation of keratin cytoskeleton into a vimentin, and cell morphologic changes. During EMT process, cells exist in three different states: epithelial (E) state, intermediate (E/M) state, and mesenchymal (M) state (**Figure 2**). As cells transition from epithelial state to mesenchymal state, they sequentially lose apical-basal polarity and cell-cell adhesion, gain anterior-posterior polarity and enhanced cell-matrix adhesions. As a result, they acquire increased abilities of migration, invasion, anti-apoptosis and extracellular matrix degradation (54, 55). The scratch assays have demonstrated that hAECs undergo EMT to migrate, further promote cell proliferation and would healing (56).

Interestingly, the same mechanism and phenomenon have also been revealed *in vivo*. Previous studies indicated that there is an age-related inflammation caused cellular senescence of hAECs. Especially when the gestation is closed to the termination, the inflammatory environments will promote the amniotic cell senescence and the happening of an irreversible EMT, leading to the loss of amniotic structure integrity and fetus delivery, which further reconfirmed the importance of EMT during the proliferation and senescence of hAECs (57, 58).

## 4 Current landscape of cell therapies

Stem cell-based therapy has recently emerged as a key player in regenerative medicine. The commonly used cell types include pluripotent stem cells, extraembryonic tissue-derived cells and adult stem cells. Among those stem cell-based clinical applications, hematopoietic stem cells (HSCs) transplantation takes the leading position, and the second place goes to mesenchymal stem cell (MSC)-based studies. Autologous or allogeneic HSC transplantation is a revolutionary life-saving procedure after irradiation or chemotherapy. The first bone marrow transplantation was reported in 1957 and it currently has become the most effective treatment for leukemia. However, HSCs extraction requires the invasive procedures to human bodies. More severely, post-operative complications are often occurred to patients. As per the statistic study of a retrospective analysis over 11 years after HSCs transplantation, of all patients, 74.2% suffered of early or late compilations, including infections, graft-versus-host disease (GVHD), CNS disorders and cardio-vascular complications, etc (59).

Human pluripotent stem cells (hPSCs) are defined as self-renewable cell types conferring the ability to differentiate into various cellular phenotypes of the human body, including three germ layers, and have gained significant interest and attention in regenerative medicine field. Multiple clinical trials using hPSC-derived cells have been launched (*clinicaltrials.gov*). The therapeutic potential of hPSCs is tremendous, but there are still some challenges that need to be overcome. One of them is the teratoma formation because of their potential for infinite proliferation. Another challenge is the need for standardization. hPSCs, especially iPSCs, are greatly heterogeneous due to their origins and preparation methods. On that account, a set of critical quality procedures and evaluation systems have to be established.

MSCs are multipotent progenitor cells possessing self-renewal ability (limited *in vitro*) and differentiation potential into mesenchymal lineages, according to the International Society for Cell and Gene Therapy (ISCT). To date, a total of 12 MSCs products have received regulatory approval for commercial use worldwide, including autologous and allogenic, to be used for spinal cord injury, osteoarthritis, GVHD, acute myocardial infarction, and Crohn’s disease. MSCs are commonly derived from umbilical cord blood, bone marrow, and adipose tissue. The cell sources are relatively limited, and their cell isolation requests higher technical skills.

**Figure 3:**
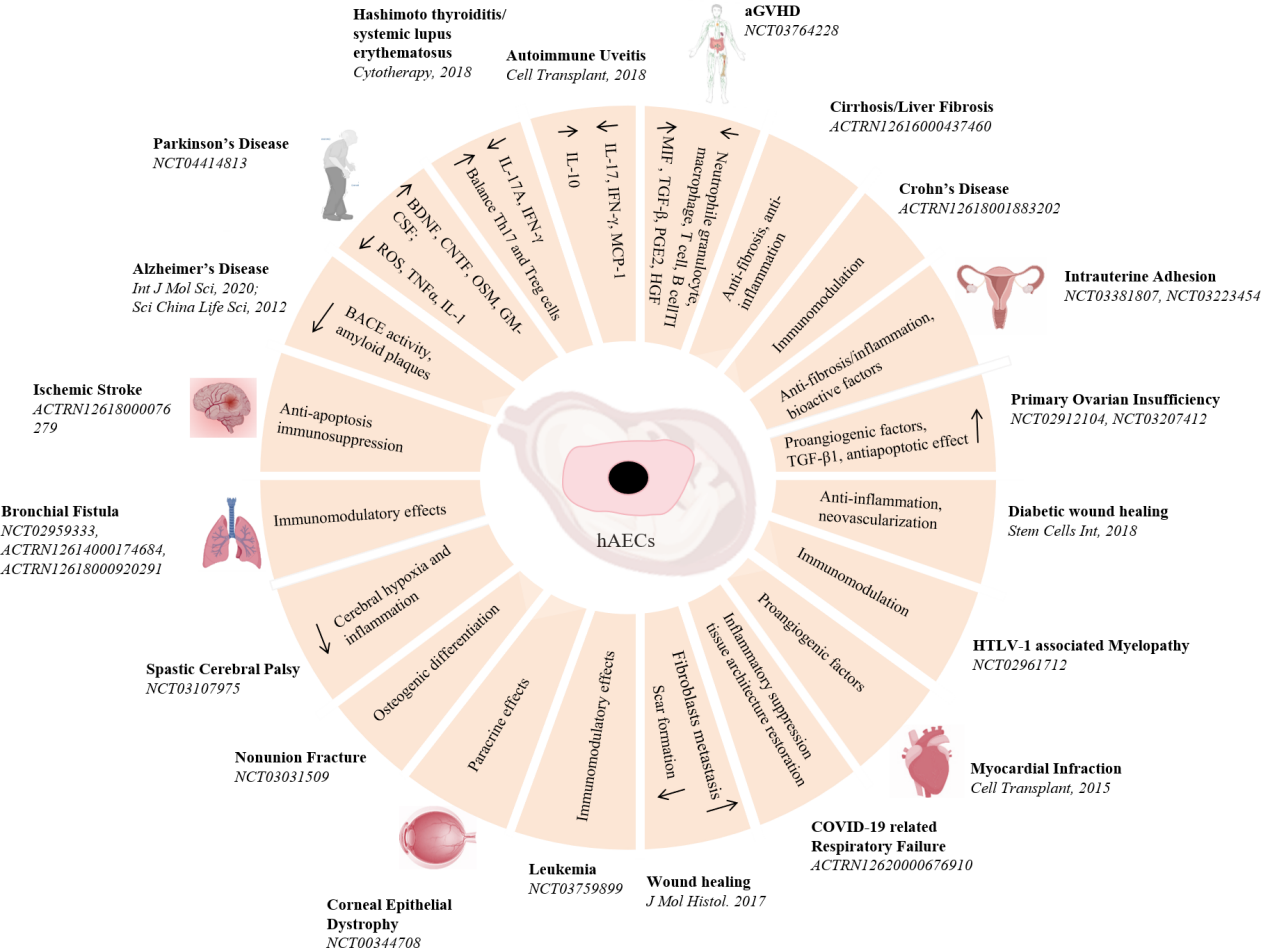
Pre-clinical and clinical studies of hAECs on diseases treatment. A schematic overview of hAECs on various diseases treatment and the underlying potential mechanisms.

Compare to all the stem cells motioned above, hAECs are morphological epithelial cells, possessing low immunogenicity, immunomodulatory and anti-inflammatory effects. Moreover, hAECs lack the telomerase activity and the potential for sustainable cell divisions, making them have a low risk of tumorigenicity. Consequently, hAECs have been considered a promising candidate for cell therapy (**Figure 3**). Despite of some uncertainty about the molecular mechanisms by which hAECs act effective on diseases, whether they are *in vivo* engrafted and differentiated or they modulate the biochemical reactions and cellular responses to injuries, the work of Wallace team has clear demonstrated that hAECs can exert a reparative effect without the need for engraftment or differentiation (60). They suggested that the primary mechanism of hAECs for lung injury repair was likely to be paracrine signaling to the surrounding tissues to reduce proinflammatory and profibrotic mediators. Similar results were also found in the studies in brain ischemia, Parkinson’s disease, spinal cord injury and wound healing (61–64). Furthermore, the transcriptome sequencing results have revealed that hAECs transplantation lead to the upregulation of several angiogenesis and inflammation molecules, such as interferon regulatory factor 7 (IRF7), Mx dynamin-like GTPase 1 (Mx1), vascular endothelial growth factor receptor 1 (VEGFR1) and VEGFR2 (65). Coculture of hAECs and freshly isolated human blood neutrophils significantly attenuated the level of oxidative burst of neutrophils (66), but directly inhibited the proliferation of naïve CD4 T cells and the production of Th1 and Th17 cytokines (67). All these cellular properties have led hAECs ideal candidate cells for various disease cell therapies.

## 5 Studies of hAECs “From Bench To Bedside”

In 1981. *Akle, C.A.*, et al. firstly isolated primary hAECs and transplanted subcutaneously into the upper arms of 7 volunteers. Four weeks later, it was observed that none of the participants exhibited detectable HLA antibodies in their blood samples, and no lymphocyte reaction was shown in 2 of the participants, indicating the transplantation of hAECs would not cause acute immune rejection (34). Since then, cell therapy using hAECs has become an emerging treatment for diseases (68–70). Since then many studies have shown satisfactory effects of hAECs in the pre-clinical treatments, however, very little outcomes were revealed on the clinical research (71, 72). As of January 2024, there are 25 clinical trials listed in the database (ClinicalTrials.gov, Anzctr.org.au, etc.) using “human amniotic epithelial cells” as biological interventions for the treatments of various diseases (**Table 3**), most promising include neurological disorders, immune diseases and tissue repairs.

**Table 3:** Summarized registrations of clinical trials utilizing human amniotic epithelial cells as biological interventions.

Category	Registration Number	Disease	Nation
Ophthalmology	NCT00344708	Corneal Epithelial Dystrophy	United States
Gynecology	NCT02912104	Primary Ovarian Insu ciency	China
	NCT03223454	Asherman’s Syndrome 1	China
	NCT03207412	Premature Ovarian Failure	China
	NCT03381807	Intrauterine Adhesion	China
	NCT04676269	Thin Endometrium Infertile	Indonesia
Neurology	NCT02961712	HTLV-1 Associated Myelopathy	China
	NCT03107975	Spastic Cerebral Palsy 1	China
	NCT04414813	Parkinson’s Disease	China
	NCT05435755	Parkinson’s Disease	China
	NCT05691114	Parkinson’s Disease	China
	ACTRN12618000076279	Ischemic Stroke	Australia
	ACTRN12622000588796	Ischemic Stroke	Australia
Pneumology	NCT02959333	Bronchial Fistula	China
	ACTRN12614000174684	Bronchopulmonary Dysplasia	Australia
	ACTRN12618000920291	Bronchopulmonary Dysplasia, Extremely Preterm Birth	Australia
	ACTRN12620000676910	COVID-19-Related Respiratory Failure	Australia
Orthopedics	NCT03031509	Nonunion Fracture	China
Others	NCT03764228	Acute Graft-Versus-Host Disease	China
	ChiCTR2000039821	Acute Graft-Versus-Host Disease	China
	NCT06164288	Acute Graft-Versus-Host Disease	China
	NCT03759899	Allogeneic Hematopoietic Stem Cell Transplantation	China
	ACTRN12616000437460	Cirrhosis, Liver Fibrosis	Australia
	ACTRN12618001883202	Crohn’s Disease, Perianal Fistulas	Australia
	NCT04728906	Myocardial Infarction	Indonesia

### 5.1 Neurological disorders

Many studies have shown that hAECs have certain biochemical characteristics of neurons and, although suspicious, are of the potential to differentiate into neural cells (26, 73). Most common accepted perspectives of hAECs in the treatment of neurologic diseases are associated with their paracrine signals. hAECs are proven to be capable of synthesizing and secreting neurotrophic factors, growth factors and neurotransmitters such as catecholamine and dopamine, which are functional in promoting the regeneration of damaged neural cells (74, 75). hAECs also secrete anti-inflammatory factors, contributing to reducing neuroinflammation, improving the cellular microenvironment, and alleviating the progressive course of diseases (64, 76). Mounting evidence has shown that hAECs administration in the animal model of neurological diseases can reduce cell apoptosis, repair damaged neurons, and re-establish damaged neural connections (77–81). It is suggested that hAECs could be a promising candidate for cell-based therapy of neurological diseases. Here, we will focus on the studies of hAECs in Parkinson’s disease and Alzheimer’s disease.

#### 5.1.1 Parkinson’s disease

Parkinson’s disease (PD) is an age-related neurodegenerative disorder. To date, PD is still incurable, and the currently available clinical treatments aim to slow down the course of disease progression and alleviate the motor symptoms. In the past decades, besides the traditional treatments of medication and surgery, an increasing number of novel therapies have been used for PD. The cell therapies have played an important role in those therapies and shown remarkable outcomes.

Many animal studies have illustrated the promising therapeutic potential of hAECs for PD treatment. In the 1990s, *Kakishita* transplanted hAECs into the striatum of PD rats and found that hAECs were able to alleviate the rat’s motor deficits, prevent the loss of dopaminergic neurons in substantia nigra pars compacta (SNpc) (82, 83). It has also been revealed that hAECs can effectively prevent the loss of TH-positive cells and dopamine in SNpc when they were transplanted into PD rats, along with ameliorating the behavioral deficits (84, 85). Studies carried out by *Zhang* et al. revealed that hAECs stereotactic transplantation into PD mice striatum facilitated the outgrowth of neurites and axonal fibers and inhibited the apoptosis of damaged dopaminergic neurons, contributing to the maintenance of the biological function of neuronal cells (64). Meanwhile, hAECs conditional medium could also improve the outgrowth of neurite *in vitro*, suggesting that hAECs might enhance the self-repair of neural cells through secreting neurotrophic factors.

Although many studies have shown that hAECs are able to relieve PD symptoms, the underlying mechanisms are not yet clearly understood. Neurotrophic factors, such as Brain-derived neurotrophic factor (BDNF), Glial cell line-derived neurotrophic factor (GDNF), Ciliary neurotrophic factor (CNTF), Oncostatin M (OSM) and Granulocyte-macrophage colony-stimulating factor (GM-CSF), secreted by hAECs, have been illustrated to promote the survival and regeneration of neural cells in the aspects of neurites outgrowth, axonal growth, synaptic plasticity, and neural cell phenotype (86, 87). Besides, hAECs were also considered to be beneficial to neuroinflammation, another main pathological feature of PD. The postmortem examinations of PD patients showed that microglia and astrocytes in the midbrain were highly activated, and a large amount of pro-inflammatory factors were detected (88, 89). Similar results were also found in PD animal models. hAECs grafts could reduce the occurrence of neuroinflammation by inhibiting microglia activation and lowering the level of TNFα and IL-1β in the PD mice striatum, additionally, hAECs secreted IL-1ra, one of the IL-1 receptor antagonists, involving in the regulation of neuroinflammation (64).

Many studies suggested that apoptosis and neuroinflammation would result into the production of oxidative stress in neural cells, further impairing neurons and exacerbating the disease progression (90, 91). Excessive amount of reactive oxygen species (ROS) was detected in substantia nigra region of PD mice, strikingly, hAECs transplantation significantly reduced the production of ROS in SNpc. In addition, the co-culture of hAECs also distinctly decreased the intracellular ROS of isolated neural cells from the midbrain of PD mice. Conversely, the intracellular ROS in hAECs levels were elevated, suggesting that hAECs to some extent might be resistant to oxidative stress (64). These studies provide a viewpoint that hAECs are promising to be a novel therapeutic approach for PD. However, more studies are still needed to reveal the specific molecular mechanisms of hAECs for the treatment.

#### 5.1.2 Alzheimer’s disease

Alzheimer’s disease (AD) is an age-related progressive neurodegenerative disorder associated with memory loss and cognitive impairment. The pathogenesis and causes of AD have not been fully understood. The prevailing proposal in the field suggests that the progressive accumulation of Amyloid beta (Aβ) might trigger a complicated cascade of reactions eventually leading to nerve death, synaptic deficits, and cholinergic neurotransmitter loss, which is widely known as “amyloid hypothesis” (92, 93). Aβ is produced through the proteolytic processing of amyloid precursor protein (APP) by β- and γ-secretases. The misfolding of the extracellular Aβ protein forming senile plaques, concomitant with the intracellular deposition of misfolded tau protein in neurofibrillary tangles cause memory loss and confusion and result in personality and cognitive decline over time (92). There is no definitive cure for AD yet, and the current available therapeutic interventions could not reverse, or even stop, the progressive course of AD.

Similar as it is for PD, hAECs have shown a good therapeutic effect on animal models of AD. It has been reported that hAECs transplantation into the lateral ventricle of AD mice models (Tg transgenic mice) via either stereotactic injection or IV injection could significantly improve their behavioral performance, along with the attenuated spatial memory deficits, the growth of cholinergic neurons in the hippocampal region of the forebrain, and the elevation of acetylcholine production. Furthermore, hAECs transplantation reduced the plaques formed by Aβ deposition and lowered the beta-secretase enzyme (BACE) activity, which is critical in regulating the production of Aβ40 and Aβ42 (94, 95). A ChIPseq analysis has demonstrated that hAECs treated with verbenalin would highly express AD- related gene sets and the genes involved in neurogenesis (96). Verbenalin has neuroprotective effects against Aβ induced neurotoxicity and has sleep-promoting and antioxidant effects (96). Similarly, hAECs in combination with lycopene (LYCO) could effectively reduce the neuroinflammatory factors, such as TNF-α and IL-1β, in cerebrospinal fluid and hippocampus tissue of AD rats, concomitant with the increased level of anti-inflammatory factors, IL-10 and TGF-β1. This united therapy significantly improved the cognitive impairments of AD rats. Importantly, it inhibited the upregulation of TLR4 and NF-κB caused by Aβ1-42 in the choroid plexus. TLR4 and NF-κB could affect the immune regulatory ability of the choroid plexus (97). These studies indicated that hAECs would be a promising candidate drug for AD treatment.

### 5.2 Immunological disorders

hAECs have immunomodulatory properties. They express low levels of major histocompatibility complex (MHC) class I surface antigens and barely express MHC class II antigens or costimulatory molecules, such as CD80 (B7-1), CD86 (B7-2), and CD40, regardless of the presence or absence of interferon gamma (IFN-γ). hAECs express neither programmed cell death receptor 1 (PD-1), an inhibitory receptor normally expressed on activated T and B cells, nor its two ligands, programmed death ligands 1 and 2 (PD-L1, PD-L2) under IFN-γ stimulation (30). Moreover, the expressions of immune inhibitory receptors, immunoglobulin-like transcript receptors 2, 3, and 4 (ILTR-2, ILTR-3, and ILTR-4) were also undetectable (30), indicating hAECs might be tolerogenic to immunological rejection. Therefore, hAECs are expected to expand their therapeutic potential in clinical applications of immunological diseases. In our previous work, transplantation of hAECs into the acute GVHD mouse models, which were established through injecting human PBMC in NCG mice, significantly reduced the infiltration of inflammatory cells into target organs and organ lesions, improved the mice survival rate, and prolonged their life span. Based on these animal studies, in 2022, the first application of Investigation of New Drug (IND) of hAECs for aGVHD treatment was approved by Center for Drug Evaluation (CDE), National Medical Products Administration (NMPA) in China and a phase I clinical trial was initiated in Nov. 2023. The outcome of this phase I clinical study will enforce the application potential of hAECs for the treatment of other immunological disorders.

Numerous studies have demonstrated the diverse immunomodulatory and anti-inflammatory properties of hAECs and hAECs conditional medium (hAECs-CM). They secrete various anti-inflammatory factors, such as MIF, TGF-β, IL-10, and PGE2, therefore, hAECs can effectively inhibit T cell proliferation and activation, reducing pro-inflammatory cytokines productions (31, 67). Furthermore, hAECs prevent the differentiation of monocyte to dendritic cells through cell direct-contact, and significantly decrease the oxidative stress of neutrophils (98). Similarly, hAECs-CM can also inhibit the chemotactic activity of neutrophils and reduce the proliferation of T cells and B cells after mitotic stimulation (66).

hAECs have been employed in a variety of immune-mediated inflammatory diseases, such as autoimmune uveitis, Hashimoto’s thyroiditis, systemic lupus erythematosus, diabetes, and Multiple sclerosis (MS) (99–102). Transplantation of hAECs inhibited myelin oligodendrocyte glycoprotein (MOG)-induced experimental autoimmune encephalomyelitis (EAE), an animal model widely used to study the pathogenesis of MS by inducing symptoms of paresthesia and CNS demyelination associated with perivascular single nucleated cell infiltration. TGF-β and PGE2 secreted by hAECs could effectively inhibit the proliferation of splenocytes. Notably, splenocytes isolated from hAECs-treated mice generated more IL-5 than the untreated group. These results indicate that hAECs may treat MS through their immunosuppression effects (103). Intradermal injection of hAECs into diabetic mice significantly accelerated diabetic wound healing and granulation tissue formation, in the meanwhile, hAECs could modulate macrophage phenotype toward M2 macrophage, promote switch from proinflammatory status to pro-healing status of wounds, and increase capillary density in diabetic wounds, which suggested hAECs could promote diabetic wound healing, at least partially, through paracrine effects to regulate inflammation and promote neovascularization (104).

### 5.3 Tissue injuries and repairing

With respect to hAECs biological functions, that is to prevent the fetus from mechanical damages and to secrete hormones and factors, supporting embryonic development, hAECs might also be favorable to tissue repair. The studies about the use of hAECs in tissues repair are abundant, including the injuries in uterus, ovary, kidney, cornea, liver, and lung. Clinical research about hAECs as biological interventions in tissue repair is also ongoing in the areas of intrauterine adhesion (NCT03381807), Asherman’s syndrome (NCT03223454), primary ovarian insufficiency/premature ovarian failure/Infertility (NCT02912104, NCT03207412), bronchial fistula (NCT02959333), spastic cerebral palsy (NCT03107975), and non-union fracture (NCT03031509). In a clinical study of persistent corneal epithelial defects (PEDs), hAECs have been found to promote the regression of PEDs when in combination with collagen shields. Complete resolution of PEDs was seen after two cycles of hAECs-seeded collagen shield in one case, and four cycles in two cases, from 7 to 12 weeks following treatment in all patients. No loss of visual acuity was reported, and clinical improvement was maintained in all cases, with a mean follow-up of 6.3 months (105).

Animal studies have been further conducted to expand the therapeutic application potential of hAECs in other tissue injuries. hAECs transplantation has been reported to functionally repair the uterine injury and collagen degradation of the scar. Following transplantation into the rats with uterine scars, hAECs induced the upregulation of VEGF1 and matrix metalloproteinase-8 (MMP-8), which facilitated angiogenesis and collagen degradation, respectively. Moreover, hAECs promoted the recovery of myometrium and endometrium (106), as well as improved the condition of intrauterine adhesion (IUA) in rats and mice models. These results showed hAECs effectively restored the facts of pregnancy and the number of fetuses, additionally, they increased the endometrial thickness and endometrial glands, reconstituted capillary regeneration, promoted stromal cell proliferation and reduced tissue fibrosis (107, 108).

In the treatment of primary ovarian insufficiency (POI), hAECs were found having an effect on regulating steroid biosynthesis and follicular development, promoting angiogenesis and reducing inflammation. In the rat models, post hAECs transplantation, the irregular estrous cycles tended to be normal, follicle stimulating hormone (FSH) level was decreased while anti-Mullerian hormone (AMH) and the count of mature follicles were increased, and rats’ body weights and ovaries sizes were also raised (65). It has been demonstrated that intravenously injected hAECs in mice with chemotherapy-induced ovarian damage were able to migrate to damaged locations and differentiated into granulosa cells, facilitating the recovery of follicle generation (109, 110).

Interestingly, hAECs were found to be able to suppress the systemic inflammation and maintain renal endothelial integrity in septic mice (111). Therefore, systemic administration of hAECs can improve mortality and renal function in ischemic-reperfusion injury-induced acute kidney injury (IRI-AKI) mice and reduce the number of apoptotic cells (112, 113). hAECs have nephroprotective effects against cisplatin-induced acute kidney injury (cisplatin-AKI) without compromising the anti-tumor activity of cisplatin (114).

Other than the applications in the conditions above, hAECs have exhibited therapeutic potentials to myocardial infarction (115, 116), cerebral hemorrhage (117), retinal degeneration (118, 119), alveolar defects (120), lung injury (121), chronic liver failure (122), gland injury (123), inner ear injury (124), and more. All these are required for future clinical research before their transformation into a therapeutic product to help the patients.

## 6 Conclusions and future challenges

Despite recent advances in stem cell-based therapies for various disease, including ESCs, iPSCs, and MSCs, there are many unclear questions and unsolved issues regarding to their potency, stability, oncogenicity, immune response, cell sources, and ethics. The major concerns for their therapeutic potentials are the high risk of tumorigenicity and invasive extraction procedures. hAECs are derived from the human amnion, which as a medical waste are readily available and under less ethical dispute, and have no tumorigenic and low immunogenic potential. The applications of hAECs on various diseases have been studied and relevant underlying mechanisms have also been explored, making them a better alternative cell source for diseases.

Although hAECs have exhibited a good efficacy in various clinical studies, their limited intrinsic capacity of proliferation lets the expansion *in vitro* and industrial manufacture challenging. Another common question about hAECs in clinical applications is the shortness of comprehensive knowledge of cell properties. Therefore, cell quality assessment for therapeutic effectiveness could not be controlled, which might lead to inconsistent outcomes in treatments.

These issues can be addressed by establishing optimized *in vitro* expansion strategies. Simultaneously, it is crucial to establish a complete and standardized evaluation system for cell properties and qualities. In the studies of hAECs lineage development and therapeutical mechanisms, by adopting new analytical methods such as single-cell transcriptome sequencing, machine learning, etc. Numerous research has looked into the deep insights of hAECs composition, as well as, cell fates and attributes during development, fundamentally solving the challenges in developing cell therapy products. On the other hand, by exploring new methods, such as combined therapy, and gene editing, the therapeutic potential of hAECs has been enhanced. In future research, the exploration of the functional features of hAECs will be the prime work to better target the disease treatment.

## Glossary

AME-E: amniotic epithelial cells-early
AME-L: amniotic epithelial cells-late
AD: Alzheimer’s disease
AMH: anti-Mullerian hormone
BDNF: brain-derived neurotrophic factor
BM-MSCs: bone marrow-derived mesenchymal stem cells
BACE: beta-secretase enzyme
CDE: Center for Drug Evaluation
CNTF: ciliary neurotrophic factor
ESCs: embryonic stem cells
EMT: epithelial mesenchymal transition
pEMT: partial epithelial mesenchymal transition
cEMT: complete epithelial mesenchymal transition
EAE: experimental autoimmune encephalomyelitis
EMP: epithelial-mesenchymal plasticity
FSH: follicle stimulating hormone
GDNF: glial-cell-line-derived neurotrophic factor
GM-CSF: granulocyte-macrophage colony-stimulating factor
GVHD: graft-versus-host disease
GCLP: good clinical laboratory practice
hAECs: human amniotic epithelial cells
hAECs-CM: hAECs conditional medium
HSCs: hematopoietic stem cells
hAMSCs: human amniotic membrane mesenchymal stem cells
HGF: hepatocyte growth factor
HSCs: hematopoietic stem cells
iPSCs: induced pluripotent stem cells
ICM: inner cell mass
IFN-γ: interferon gamma
IL10: interleukin-10
ILTR-2: immunoglobulin-like transcript receptors 2
ILTR-3: immunoglobulin-like transcript receptors 3
ILTR-4: immunoglobulin-like transcript receptors 4
IND: Investigation of New Drug
IUA: Intrauterine adhesion
IRF7: interferon regulatory factor 7
IRI-AKI: ischemic-reperfusion injury-induced acute kidney injury
LYCO: lycopene
MIF: macrophage migration inhibitory factor
MHC: major histocompatibility complex
MOG: myelin oligodendrocyte glycoprotein
MS: multiple sclerosis
MSC: mesenchymal stem cell
Mx1: Mx dynamin-like GTPase 1
NMPA: National Medical Products Administration
OSM: Oncostatin M
PSCs: pluripotent stem cells
PBMC: peripheral blood mononuclear cell
PGE2: prostaglandin E2
PD: Parkinson’s disease
PD-1: programmed cell death receptor 1
PD-L1: programmed death ligands 1
PD-L2: programmed death ligands 2
POI: primary ovarian insufficiency
PEDs: persistent corneal epithelial defects
μPASE: microfluidic platform for analysis of single embryos
ROS: reactive oxygen species
scRNA-seq: single-cell transcriptome sequencing
SNpc: substantia nigra pars compacta
TE: trophectoderm
TGF-β: transforming growth factor-β
VEGFR1: vascular endothelial growth factor receptor 1

## References

[1] Parolini O Alviano F Bagnara GP Bilic G Buhring HJ Evangelista M. Concise review: isolation and characterization of cells from human term placenta: outcome of the first international Workshop on Placenta Derived Stem Cells. Stem Cells. (2008) 26:300–11. doi: 10.1634/stemcells.2007-0594 17975221

[2] Miki T Marongiu F Dorko K Ellis EC Strom SC. Isolation of amniotic epithelial stem cells. Curr Protoc Stem Cell Biol. (2010) 3. doi: 10.1002/9780470151808.sc01e03s12. *Chapter 1*, Unit 1E. 20049689

[3] Izumi M Pazin BJ Minervini CF Gerlach J Ross MA Stolz DB. Quantitative comparison of stem cell marker-positive cells in fetal and term human amnion. J Reprod Immunol. (2009) 81:39–43. doi: 10.1016/j.jri.2009.02.007 19501410

[4] Johnson MH McConnell JM. Lineage allocation and cell polarity during mouse embryogenesis. Semin Cell Dev Biol. (2004) 15:583–97. doi: 10.1016/j.semcdb.2004.04.002 15271304

[5] Sutherland AE Speed TP Calarco PG. Inner cell allocation in the mouse morula The role of oriented division during fourth cleavage. Dev Biol. (1990) 137:13–25. doi: 10.1016/0012-1606(90)90003-2 2295360

[6] Shahbazi MN Zernicka-Goetz M. Deconstructing and reconstructing the mouse and human early embryo. Nat Cell Biol. (2018) 20:878–87. doi: 10.1038/s41556-018-0144-x 30038253

[7] Nakamura T Okamoto I Sasaki K Yabuta Y Iwatani C Tsuchiya H. A developmental coordinate of pluripotency among mice, monkeys and humans. Nature. (2016) 537:57–62. doi: 10.1038/nature19096 27556940

[8] Gopalakrishna A Karim KB. Fetal membranes and placentation in Chiroptera. J Reprod Fertil. (1979) 56:417–29. doi: 10.1530/jrf.0.0560417 381657

[9] Rostovskaya M Andrews S Reik W Rugg-Gunn PJ. Amniogenesis occurs in two independent waves in primates. Cell Stem Cell. (2022) 29:744–759 e746. doi: 10.1016/j.stem.2022.03.014 35439430 PMC9627701

[10] Pereira PN Dobreva MP Graham L Huylebroeck D Lawson KA Zwijsen AN. Amnion formation in the mouse embryo: the single amniochorionic fold model. BMC Dev Biol. (2011) 11:48. doi: 10.1186/1471-213X-11-48 21806820 PMC3163621

[11] Dobreva MP Abon Escalona V Lawson KA Sanchez MN Ponomarev LC Pereira PNG. Amniotic ectoderm expansion in mouse occurs via distinct modes and requires SMAD5-mediated signalling. Development. (2018) 145. doi: 10.1242/dev.157222 PMC6053664 29884675

[12] Takashima S Yasuo M Sanzen N Sekiguchi K Okabe M Yoshida T. Characterization of laminin isoforms in human amnion. Tissue Cell. (2008) 40:75–81. doi: 10.1016/j.tice.2007.09.001 18291433

[13] Whittle WL Gibb W Challis JR. The characterization of human amnion epithelial and mesenchymal cells: the cellular expression, activity and glucocorticoid regulation of prostaglandin output. Placenta. (2000) 21:394–401. doi: 10.1053/plac.1999.0482 10833375

[14] Liu QW Huang QM Wu HY Zuo GS Gu HC Deng KY. Characteristics and therapeutic potential of human amnion-derived stem cells. Int J Mol Sci. (2021) 22:970. doi: 10.3390/ijms22020970 33478081 PMC7835733

[15] Carlson B. Formation of germ layers and early derivatives. Hum embryol Dev Biol. (2014), 75–91. doi: 10.1016/b978-1-4557-2794-0.00005-x

[16] Nichols J Smith A. Naive and primed pluripotent states. Cell Stem Cell. (2009) 4:487–92. doi: 10.1016/j.stem.2009.05.015 19497275

[17] Shao Y Taniguchi K Gurdziel K Townshend RF Xue X Yong KMA. Self-organized amniogenesis by human pluripotent stem cells in a biomimetic implantation-like niche. Nat Mater. (2017) 16:419–25. doi: 10.1038/nmat4829 PMC5374007 27941807

[18] Yu L Wei Y Duan J Schmitz DA Sakurai M Wang L. Blastocyst-like structures generated from human pluripotent stem cells. Nature. (2021) 591:620–6. doi: 10.1038/s41586-021-03356-y 33731924

[19] Liu X Tan JP Schroder J Aberkane A Ouyang JF Mohenska M. Modelling human blastocysts by reprogramming fibroblasts into iBlastoids. Nature. (2021) 591:627–32. doi: 10.1038/s41586-021-03372-y 33731926

[20] Zhu Y Wang H Yin F Guo Y Li F Gao D. Amnion-on-a-chip: modeling human amniotic development in mid-gestation from pluripotent stem cells. Lab Chip. (2020) 20:3258–68. doi: 10.1039/d0lc00268b 32749421

[21] Barboni B Russo V Curini V Martelli A Berardinelli P Mauro A. Gestational stage affects amniotic epithelial cells phenotype, methylation status, immunomodulatory and stemness properties. Stem Cell Rev Rep. (2014) 10:725–41. doi: 10.1007/s12015-014-9519-y PMC4167432 24867872

[22] Zhu D Kusuma GD Schwab R Chan ST Tan J Saad MI. Prematurity negatively affects regenerative properties of human amniotic epithelial cells in the context of lung repair. Clin Sci (Lond). (2020) 134:2665–79. doi: 10.1042/CS20200859 33000862

[23] Gaggi G Di Credico A Izzicupo P Antonucci I Crescioli C Di Giacomo V. Epigenetic features of human perinatal stem cells redefine their stemness potential. Cells. (2020) 9. doi: 10.3390/cells9051304 PMC7290760 32456308

[24] Miki T Strom SC. Amnion-derived pluripotent/multipotent stem cells. Stem Cell Rev. (2006) 2:133–42. doi: 10.1007/s12015-006-0020-0 17237552

[25] Miki T Mitamura K Ross MA Stolz DB Strom SC. Identification of stem cell marker-positive cells by immunofluorescence in term human amnion. J Reprod Immunol. (2007) 75:91–6. doi: 10.1016/j.jri.2007.03.017 17493686

[26] Miki T Lehmann T Cai H Stolz DB Strom SC. Stem cell characteristics of amniotic epithelial cells. Stem Cells. (2005) 23:1549–59. doi: 10.1634/stemcells.2004-0357 16081662

[27] Adinolfi M Akle CA McColl I Fensom AH Tansley L Connolly P. Expression of HLA antigens, beta 2-microglobulin and enzymes by human amniotic epithelial cells. Nature. (1982) 295:325–7. doi: 10.1038/295325a0 6173762

[28] Hammer A Hutter H Blaschitz A Mahnert W Hartmann M Uchanska-Ziegler B. Amnion epithelial cells, in contrast to trophoblast cells, express all classical HLA class I molecules together with HLA-G. Am J Reprod Immunol. (1997) 37:161–71. doi: 10.1111/j.1600-0897.1997.tb00208.x 9083612

[29] Strom SC Gramignoli R. Human amnion epithelial cells expressing HLA-G as novel cell-based treatment for liver disease. Hum Immunol. (2016) 77:734–9. doi: 10.1016/j.humimm.2016.07.002 27476049

[30] Insausti CL Blanquer M Garcia-Hernandez AM Castellanos G Moraleda JM. Amniotic membrane-derived stem cells: immunomodulatory properties and potential clinical application. Stem Cells Cloning. (2014) 7:53–63. doi: 10.2147/SCCAA.S58696 24744610 PMC3969346

[31] Li H Niederkorn JY Neelam S Mayhew E Word RA McCulley JP. Immunosuppressive factors secreted by human amniotic epithelial cells. Invest Ophthalmol Vis Sci. (2005) 46:900–7. doi: 10.1167/iovs.04-0495 15728546

[32] Kubo M Sonoda Y Muramatsu R Usui M. Immunogenicity of human amniotic membrane in experimental xenotransplantation. Invest Ophthalmol Vis Sci. (2001) 42:1539–46. 11381058

[33] Bailo M Soncini M Vertua E Signoroni PB Sanzone S Lombardi G. Engraftment potential of human amnion and chorion cells derived from term placenta. Transplantation. (2004) 78:1439–48. doi: 10.1097/01.tp.0000144606.84234.49 15599307

[34] Akle CA Adinolfi M Welsh KI Leibowitz S McColl I. Immunogenicity of human amniotic epithelial cells after transplantation into volunteers. Lancet. (1981) 2:1003–5. doi: 10.1016/s0140-6736(81)91212-5 6118474

[35] Motedayyen H Esmaeil N Tajik N Khadem F Ghotloo S Khani B. Method and key points for isolation of human amniotic epithelial cells with high yield, viability and purity. BMC Res Notes. (2017) 10:552. doi: 10.1186/s13104-017-2880-6 29096713 PMC5669002

[36] Gramignoli R Srinivasan RC Kannisto K Strom SC. Isolation of human amnion epithelial cells according to current good manufacturing procedures. Curr Protoc Stem Cell Biol. (2016) 37:1E 10 11–11E 10 13. doi: 10.1002/cpsc.2 27171794

[37] Weidinger A Pozenel L Wolbank S Banerjee A. Sub-regional differences of the human amniotic membrane and their potential impact on tissue regeneration application. Front Bioeng Biotechnol. (2020) 8:613804. doi: 10.3389/fbioe.2020.613804 33520964 PMC7839410

[38] Ghamari SH Abbasi-Kangevari M Tayebi T Bahrami S Niknejad H. The bottlenecks in translating placenta-derived amniotic epithelial and mesenchymal stromal cells into the clinic: current discrepancies in marker reports. Front Bioeng Biotechnol. (2020) 8:180. doi: 10.3389/fbioe.2020.00180 32232037 PMC7083014

[39] Yang PJ Yuan WX Liu J Li JY Tan B Qiu C. Biological characterization of human amniotic epithelial cells in a serum-free system and their safety evaluation. Acta Pharmacol Sin. (2018) 39:1305–16. doi: 10.1038/aps.2018.22 PMC6289351 29565036

[40] Murphy SV Kidyoor A Reid T Atala A Wallace EM Lim R. Isolation, cryopreservation and culture of human amnion epithelial cells for clinical applications. J Vis Exp. (2014) 94:52085. doi: 10.3791/52085 PMC4356357 25548905

[41] Naeem A Choudhry M Kroemer A Wahnschafft S Cui W Albanese C. Expansion of human amniotic epithelial cells using condition cell reprogramming technology. Hum Cell. (2023) 36:602–11. doi: 10.1007/s13577-022-00849-4 PMC9947022 36586053

[42] Zhou K Koike C Yoshida T Okabe M Fathy M Kyo S. Establishment and characterization of immortalized human amniotic epithelial cells. Cell Reprogram. (2013) 15:55–67. doi: 10.1089/cell.2012.0021 23298399 PMC3567704

[43] Guo G Stirparo GG Strawbridge SE Spindlow D Yang J Clarke J. Human naive epiblast cells possess unrestricted lineage potential. Cell Stem Cell. (2021) 28:1040–1056 e1046. doi: 10.1016/j.stem.2021.02.025 33831366 PMC8189439

[44] Tian YB Wang NX Xu Y Yu CY Liu RM Luo Y. Hyaluronic acid ameliorates the proliferative ability of human amniotic epithelial cells through activation of TGF-beta/BMP signaling. PeerJ. (2020) 8:e10104. doi: 10.7717/peerj.10104 33062456 PMC7532780

[45] Liu J Wen Y Luo W Liu Y Sha X. Human amniotic epithelial cells promote the proliferation of human corneal endothelial cells by regulating telomerase activity via the wnt/beta-catenin pathway. Curr Eye Res. (2021) 46:159–67. doi: 10.1080/02713683.2020.1792508 32631162

[46] Murphy S Rosli S Acharya R Mathias L Lim R Wallace E. Amnion epithelial cell isolation and characterization for clinical use. Curr Protoc Stem Cell Biol. (2010) 6. doi: 10.1002/9780470151808.sc01e06s13 20373516

[47] Alcaraz A Mrowiec A Insausti CL Garcia-Vizcaino EM Ruiz-Canada C Lopez-Martinez MC. Autocrine TGF-beta induces epithelial to mesenchymal transition in human amniotic epithelial cells. Cell Transplant. (2013) 22:1351–67. doi: 10.3727/096368912x657387 23031712

[48] Takano C Horie M Taiko I Trinh QD Kanemaru K Komine-Aizawa S. Inhibition of epithelial-mesenchymal transition maintains stemness in human amniotic epithelial cells. Stem Cell Rev Rep. (2022) 18:3083–91. doi: 10.1007/s12015-022-10420-1 PMC9622541 35931939

[49] Bakir B Chiarella AM Pitarresi JR Rustgi AK. EMT, MET, plasticity, and tumor metastasis. Trends Cell Biol. (2020) 30:764–76. doi: 10.1016/j.tcb.2020.07.003 PMC7647095 32800658

[50] Yang J Antin P Berx G Blanpain C Brabletz T Bronner M. Guidelines and definitions for research on epithelial-mesenchymal transition. Nat Rev Mol Cell Biol. (2020) 21:341–52. doi: 10.1038/s41580-020-0237-9 PMC7250738 32300252

[51] Verstappe J Berx G. A role for partial epithelial-to-mesenchymal transition in enabling stemness in homeostasis and cancer. Semin Cancer Biol. (2023) 90:15–28. doi: 10.1016/j.semcancer.2023.02.001 36773819

[52] Lambert AW Weinberg RA. Linking EMT programmes to normal and neoplastic epithelial stem cells. Nat Rev Cancer. (2021) 21:325–38. doi: 10.1038/s41568-021-00332-6 33547455

[53] Abell AN Jordan NV Huang W Prat A Midland AA Johnson NL. MAP3K4/CBP-regulated H2B acetylation controls epithelial-mesenchymal transition in trophoblast stem cells. Cell Stem Cell. (2011) 8:525–37. doi: 10.1016/j.stem.2011.03.008 PMC3091002 21549327

[54] Oliveira MS Barreto-Filho JB. Placental-derived stem cells: Culture, differentiation and challenges. World J Stem Cells. (2015) 7:769–75. doi: 10.4252/wjsc.v7.i4.769 PMC4444616 26029347

[55] Janzen C Sen S Lei MY Gagliardi de Assumpcao M Challis J Chaudhuri G. The role of epithelial to mesenchymal transition in human amniotic membrane rupture. J Clin Endocrinol Metab. (2017) 102:1261–9. doi: 10.1210/jc.2016-3150 PMC5460731 28388726

[56] Richardson L Menon R. Proliferative, migratory, and transition properties reveal metastate of human amnion cells. Am J Pathol. (2018) 188:2004–15. doi: 10.1016/j.ajpath.2018.05.019 PMC6119821 29981743

[57] de Castro Silva M Richardson LS Kechichian T Urrabaz-Garza R da Silva MG Menon R. Inflammation, but not infection, induces EMT in human amnion epithelial cells. Reprod (Cambridge England). (2020) 160:627–38. doi: 10.1530/REP-20-0283 32841157

[58] Richardson LS Taylor RN Menon R. Reversible EMT and MET mediate amnion remodeling during pregnancy and labor. Sci Signaling. (2020) 13:eaay1486. doi: 10.1126/scisignal.aay1486 PMC7092701 32047115

[59] Hierlmeier S Eyrich M Wölfl M Schlegel PG Wiegering V. Early and late complications following hematopoietic stem cell transplantation in pediatric patients - A retrospective analysis over 11 years. PloS One. (2018) 13:e0204914. doi: 10.1371/journal.pone.0204914 30325953 PMC6191171

[60] Murphy S Lim R Dickinson H Acharya R Rosli S Jenkin G. Human amnion epithelial cells prevent bleomycin-induced lung injury and preserve lung function. Cell Transplant. (2011) 20:909–23. doi: 10.3727/096368910x543385 21092408

[61] Sankar V Muthusamy R. Role of human amniotic epithelial cell transplantation in spinal cord injury repair research. Neuroscience. (2003) 118:11–7. doi: 10.1016/s0306-4522(02)00929-6 12676132

[62] Yu SJ Soncini M Kaneko Y Hess DC Parolini O Borlongan CV. Amnion: a potent graft source for cell therapy in stroke. Cell Transplant. (2009) 18:111–8. doi: 10.3727/096368909788341243 19499700

[63] Branski LK Herndon DN Celis MM Norbury WB Masters OE Jeschke MG. Amnion in the treatment of pediatric partial-thickness facial burns. Burns. (2008) 34:393–9. doi: 10.1016/j.burns.2007.06.007 17920202

[64] Zhang J Li H Yang H Lin J Wang Y Zhang Q. Human amniotic epithelial cells alleviate a mouse model of Parkinson's disease mainly by neuroprotective, anti oxidative and anti-inflammatory factors. J neuroimmune Pharmacol. (2021) 16:620–33. doi: 10.1007/s11481-020-09969-w 33164162

[65] Zhang Y Ouyang X You S Zou H Shao X Zhang G. Effect of human amniotic epithelial cells on ovarian function, fertility and ovarian reserve in primary ovarian insufficiency rats and analysis of underlying mechanisms by mRNA sequencing. Am J Transl Res. (2020) 12:3234–54. PMC7407690 32774697

[66] Alipour R Motedayyen H Sereshki N Rafiee M Alsahebfosul F Pourazar A. Human amniotic epithelial cells affect the functions of neutrophils. Int J Stem Cells. (2020) 13:212–20. doi: 10.15283/ijsc19155 PMC7378904 32323513

[67] Motedayyen H Rezaei A Zarnani AH Tajik N. Human amniotic epithelial cells inhibit activation and pro-inflammatory cytokines production of naive CD4+ T cells from women with unexplained recurrent spontaneous abortion. Reprod Biol. (2018) 18:182–8. doi: 10.1016/j.repbio.2018.04.002 29729842

[68] Andrewartha N Yeoh G. Human amnion epithelial cell therapy for chronic liver disease. Stem Cells Int. (2019) 2019:8106482. doi: 10.1155/2019/8106482 31485235 PMC6702811

[69] Zhang Q Lai D. Application of human amniotic epithelial cells in regenerative medicine: a systematic review. Stem Cell Res Ther. (2020) 11:439. doi: 10.1186/s13287-020-01951-w 33059766 PMC7559178

[70] Hodges RJ Lim R Jenkin G Wallace EM. Amnion epithelial cells as a candidate therapy for acute and chronic lung injury. Stem Cells Int. (2012) 709763. doi: 10.1155/2012/709763 PMC3345254 22577395

[71] Phan TG Ma H Lim R Sobey CG Wallace EM. Phase 1 trial of amnion cell therapy for ischemic stroke. Front Neurol. (2018) 9:198. doi: 10.3389/fneur.2018.00198 29930530 PMC5999782

[72] Phan TG Lim R Chan ST McDonald H Gan PY Zhang SR. Phase I trial outcome of amnion cell therapy in patients with ischemic stroke (I-ACT). Front Neurosci. (2023) 17:1153231. doi: 10.3389/fnins.2023.1153231 37229431 PMC10204798

[73] Ilancheran S Michalska A Peh G Wallace EM Pera M Manuelpillai U. Stem cells derived from human fetal membranes display multilineage differentiation potential. Biol Reprod. (2007) 77:577–88. doi: 10.1095/biolreprod.106.055244 17494917

[74] Uchida S Inanaga Y Kobayashi M Hurukawa S Araie M Sakuragawa N. Neurotrophic function of conditioned medium from human amniotic epithelial cells. J Neurosci Res. (2000) 62:585–90. doi: 10.1002/(ISSN)1097-4547 11070502

[75] Grzywocz Z Pius-Sadowska E Klos P Gryzik M Wasilewska D Aleksandrowicz B. Growth factors and their receptors derived from human amniotic cells in *vitro* . Folia Histochem Cytobiol. (2014) 52:163–70. doi: 10.5603/FHC.2014.0019 25308731

[76] Hao Y Ma DH Hwang DG Kim WS Zhang F. Identification of antiangiogenic and antiinflammatory proteins in human amniotic membrane. Cornea. (2000) 19:348–52. doi: 10.1097/00003226-200005000-00018 10832697

[77] Liu T Wu J Huang Q Hou Y Jiang Z Zang S. Human amniotic epithelial cells ameliorate behavioral dysfunction and reduce infarct size in the rat middle cerebral artery occlusion model. Shock. (2008) 29:603–11. doi: 10.1097/SHK.0b013e318157e845 18414234

[78] Teo L Bourne JA. A reproducible and translatable model of focal ischemia in the visual cortex of infant and adult marmoset monkeys. Brain Pathol. (2014) 24:459–74. doi: 10.1111/bpa.12129 PMC8029183 25469561

[79] Wu ZY Hui GZ Lu Y Wu X Guo LH. Transplantation of human amniotic epithelial cells improves hindlimb function in rats with spinal cord injury. Chin Med J (Engl). (2006) 119:2101–7. doi: 10.1097/00029330-200612020-00013 17199962

[80] Leaw B Zhu D Tan J Muljadi R Saad MI Mockler JC. Human amnion epithelial cells rescue cell death via immunomodulation of microglia in a mouse model of perinatal brain injury. Stem Cell Res Ther. (2017) 8:46. doi: 10.1186/s13287-017-0496-3 28241859 PMC5330154

[81] Wang TG Xu J Zhu AH Lu H Miao ZN Zhao P. Human amniotic epithelial cells combined with silk fibroin scaffold in the repair of spinal cord injury. Neural Regener Res. (2016) 11:1670–7. doi: 10.4103/1673-5374.193249 PMC5116849 27904501

[82] Kakishita K Elwan MA Nakao N Itakura T Sakuragawa N. Human amniotic epithelial cells produce dopamine and survive after implantation into the striatum of a rat model of Parkinson's disease: a potential source of donor for transplantation therapy. Exp Neurol. (2000) 165:27–34. doi: 10.1006/exnr.2000.7449 10964482

[83] Kakishita K Nakao N Sakuragawa N Itakura T. Implantation of human amniotic epithelial cells prevents the degeneration of nigral dopamine neurons in rats with 6-hydroxydopamine lesions. Brain Res. (2003) 980:48–56. doi: 10.1016/s0006-8993(03)02875-0 12865158

[84] Yang X Song L Wu N Liu Z Xue S Hui G. An experimental study on intracerebroventricular transplantation of human amniotic epithelial cells in a rat model of Parkinson's disease. Neurol Res. (2010) 32:1054–9. doi: 10.1179/016164110X12681290831207 20483022

[85] Yang XX Xue SR Dong WL Kong Y. Therapeutic effect of human amniotic epithelial cell transplantation into the lateral ventricle of hemiparkinsonian rats. Chin Med J (Engl). (2009) 122:2449–54. 20079158

[86] Tome D Fonseca CP Campos FL Baltazar G. Role of neurotrophic factors in Parkinson's disease. Curr Pharm Des. (2017) 23:809–38. doi: 10.2174/1381612822666161208120422 27928963

[87] Domanskyi A Saarma M Airavaara M. Prospects of neurotrophic factors for Parkinson's disease: comparison of protein and gene therapy. Hum Gene Ther. (2015) 26:550–9. doi: 10.1089/hum.2015.065 26176331

[88] Kempuraj D Thangavel R Natteru PA Selvakumar GP Saeed D Zahoor H. Neuroinflammation induces neurodegeneration. J Neurol Neurosurg Spine. (2016) 1:(1):1003. 28127589 PMC5260818

[89] Badanjak K Fixemer S Smajic S Skupin A Grunewald A. The contribution of microglia to neuroinflammation in Parkinson's disease. Int J Mol Sci. (2021) 22. doi: 10.3390/ijms22094676 PMC8125756 33925154

[90] Bjorklund G Peana M Maes M Dadar M Severin B. The glutathione system in Parkinson's disease and its progression. Neurosci Biobehav Rev. (2021) 120:470–8. doi: 10.1016/j.neubiorev.2020.10.004 33068556

[91] Bose A Beal MF. Mitochondrial dysfunction in Parkinson's disease. J Neurochem. (2016) 139 Suppl 1:216–31. doi: 10.1111/jnc.13731 27546335

[92] Karran E De Strooper B. The amyloid hypothesis in Alzheimer disease: new insights from new therapeutics. Nat Rev Drug Discovery. (2022) 21:306–18. doi: 10.1038/s41573-022-00391-w 35177833

[93] Jack CR Jr. Wiste HJ Vemuri P Weigand SD Senjem ML Zeng G. Brain beta-amyloid measures and magnetic resonance imaging atrophy both predict time-to-progression from mild cognitive impairment to Alzheimer's disease. Brain. (2010) 133:3336–48. doi: 10.1093/brain/awq277 PMC2965425 20935035

[94] Xue S Chen C Dong W Hui G Liu T Guo L. Therapeutic effects of human amniotic epithelial cell transplantation on double-transgenic mice co-expressing APPswe and PS1DeltaE9-deleted genes. Sci China Life Sci. (2012) 55:132–40. doi: 10.1007/s11427-012-4283-1 22415684

[95] Kim KY Suh YH Chang KA. Therapeutic effects of human amniotic epithelial stem cells in a transgenic mouse model of Alzheimer's disease. Int J Mol Sci. (2020) 21(7):2658. doi: 10.3390/ijms21072658 32290355 PMC7178120

[96] Ferdousi F Kondo S Sasaki K Uchida Y Ohkohchi N Zheng YW. Microarray analysis of verbenalin-treated human amniotic epithelial cells reveals therapeutic potential for Alzheimer's Disease. Aging (Albany NY). (2020) 12:5516–38. doi: 10.18632/aging.102985 PMC7138585 32224504

[97] Xu Z Liu C Wang R Gao X Hao C Liu C. A combination of lycopene and human amniotic epithelial cells can ameliorate cognitive deficits and suppress neuroinflammatory signaling by choroid plexus in Alzheimer's disease rat. J Nutr Biochem. (2021) 88:108558. doi: 10.1016/j.jnutbio.2020.108558 33249184

[98] Magatti M Caruso M De Munari S Vertua E De D Manuelpillai U. Human amniotic membrane-derived mesenchymal and epithelial cells exert different effects on monocyte-derived dendritic cell differentiation and function. Cell Transplant. (2015) 24:1733–52. doi: 10.3727/096368914X684033 25259480

[99] Tan B Yuan W Li J Yang P Ge Z Liu J. Therapeutic effect of human amniotic epithelial cells in murine models of Hashimoto's thyroiditis and Systemic lupus erythematosus. Cytotherapy. (2018) 20:1247–58. doi: 10.1016/j.jcyt.2018.04.001 30174233

[100] Li J Qiu C Zhang Z Yuan W Ge Z Tan B. Subretinal transplantation of human amniotic epithelial cells in the treatment of autoimmune uveitis in rats. Cell Transplant. (2018) 27:1504–14. doi: 10.1177/0963689718796196 PMC6180726 30168350

[101] Zou G Liu T Guo L Huang Y Feng Y Duan T. MicroRNA−32 silences WWP2 expression to maintain the pluripotency of human amniotic epithelial stem cells and beta islet−like cell differentiation. Int J Mol Med. (2018) 41:1983–91. doi: 10.3892/ijmm.2018.3436 PMC5810217 29393344

[102] Liu YH Chan J Vaghjiani V Murthi P Manuelpillai U Toh BH. Human amniotic epithelial cells suppress relapse of corticosteroid-remitted experimental autoimmune disease. Cytotherapy. (2014) 16:535–44. doi: 10.1016/j.jcyt.2013.10.007 24411589

[103] Liu YH Vaghjiani V Tee JY To K Cui P Oh DY. Amniotic epithelial cells from the human placenta potently suppress a mouse model of multiple sclerosis. PloS One. (2012) 7:e35758. doi: 10.1371/journal.pone.0035758 22563398 PMC3338525

[104] Zheng Y Zheng S Fan X Li L Xiao Y Luo P. Amniotic epithelial cells accelerate diabetic wound healing by modulating inflammation and promoting neovascularization. Stem Cells Int. (2018) 1082076. doi: 10.1155/2018/1082076 PMC6120261 30210547

[105] Parmar DN Alizadeh H Awwad ST Li H Neelam S Bowman RW. Ocular surface restoration using non-surgical transplantation of tissue-cultured human amniotic epithelial cells. Am J Ophthalmol. (2006) 141:299–307. doi: 10.1016/j.ajo.2005.09.008 16458684

[106] Fan Y Sun J Zhang Q Lai D. Transplantation of human amniotic epithelial cells promotes morphological and functional regeneration in a rat uterine scar model. Stem Cell Res Ther. (2021) 12:207. doi: 10.1186/s13287-021-02260-6 33762002 PMC7992833

[107] Li B Zhang Q Sun J Lai D. Human amniotic epithelial cells improve fertility in an intrauterine adhesion mouse model. Stem Cell Res Ther. (2019) 10:257. doi: 10.1186/s13287-019-1368-9 31412924 PMC6694540

[108] Ouyang X You S Zhang Y Zhang C Zhang G Shao X. Transplantation of human amnion epithelial cells improves endometrial regeneration in rat model of intrauterine adhesions. Stem Cells Dev. (2020) 29:1346–62. doi: 10.1089/scd.2019.0246 32772798

[109] Wang F Wang L Yao X Lai D Guo L. Human amniotic epithelial cells can differentiate into granulosa cells and restore folliculogenesis in a mouse model of chemotherapy-induced premature ovarian failure. Stem Cell Res Ther. (2013) 4:124. doi: 10.1186/scrt335 24406076 PMC3854701

[110] Zhang Q Huang Y Sun J Gu T Shao X Lai D. Immunomodulatory effect of human amniotic epithelial cells on restoration of ovarian function in mice with autoimmune ovarian disease. Acta Biochim Biophys Sin (Shanghai). (2019) 51:845–55. doi: 10.1093/abbs/gmz065 31287492

[111] Chi D Chen Y Xiang C Yao W Wang H Zheng X. Human amnion epithelial cells and their derived exosomes alleviate sepsis-associated acute kidney injury via mitigating endothelial dysfunction. Front Med (Lausanne). (2022) 9:829606. doi: 10.3389/fmed.2022.829606 35402422 PMC8989462

[112] Ren Y Chen Y Zheng X Wang H Kang X Tang J. Human amniotic epithelial cells ameliorate kidney damage in ischemia-reperfusion mouse model of acute kidney injury. Stem Cell Res Ther. (2020) 11:410. doi: 10.1186/s13287-020-01917-y 32967729 PMC7510147

[113] Liu J Hua R Gong Z Shang B Huang Y Guo L. Human amniotic epithelial cells inhibit CD4+ T cell activation in acute kidney injury patients by influencing the miR-101-c-Rel-IL-2 pathway. Mol Immunol. (2017) 81:76–84. doi: 10.1016/j.molimm.2016.11.019 27898347

[114] Kang X Chen Y Xin X Liu M Ma Y Ren Y. Human amniotic epithelial cells and their derived exosomes protect against cisplatin-induced acute kidney injury without compromising its antitumor activity in mice. Front Cell Dev Biol. (2021) 9:752053. doi: 10.3389/fcell.2021.752053 35186944 PMC8851426

[115] Song YS Joo HW Park IH Shen GY Lee Y Shin JH. Transplanted human amniotic epithelial cells secrete paracrine proangiogenic cytokines in rat model of myocardial infarction. Cell Transplant. (2015) 24:2055–64. doi: 10.3727/096368914X685609 25420194

[116] Fang CH Jin J Joe JH Song YS So BI Lim SM. *In vivo* differentiation of human amniotic epithelial cells into cardiomyocyte-like cells and cell transplantation effect on myocardial infarction in rats: comparison with cord blood and adipose tissue-derived mesenchymal stem cells. Cell Transplant. (2012) 21:1687–96. doi: 10.3727/096368912X653039 22776022

[117] Dong W Chen H Yang X Guo L Hui G. Treatment of intracerebral haemorrhage in rats with intraventricular transplantation of human amniotic epithelial cells. Cell Biol Int. (2010) 34:573–7. doi: 10.1042/CBI20090248 20184556

[118] Li J Qiu C Wei Y Yuan W Liu J Cui W. Human amniotic epithelial stem cell-derived retinal pigment epithelium cells repair retinal degeneration. Front Cell Dev Biol. (2021) 9:737242. doi: 10.3389/fcell.2021.737242 34650985 PMC8505778

[119] Li J Qiu C Zhou J Wei Y Yuan W Liu J. Repair of retinal degeneration by human amniotic epithelial stem cell-derived photoreceptor-like cells. Int J Mol Sci. (2022) 23:8722. doi: 10.3390/ijms23158722 35955866 PMC9369429

[120] Jiawen S Jianjun Z Jiewen D Dedong Y Hongbo Y Jun S. Osteogenic differentiation of human amniotic epithelial cells and its application in alveolar defect restoration. Stem Cells Transl Med. (2014) 3:1504–13. doi: 10.5966/sctm.2014-0118 PMC4250213 25368378

[121] Hodges RJ Jenkin G Hooper SB Allison B Lim R Dickinson H. Human amnion epithelial cells reduce ventilation-induced preterm lung injury in fetal sheep. Am J Obstet Gynecol. (2012) 206:448 e448–415. doi: 10.1016/j.ajog.2012.02.038 22542124

[122] Lin JS Zhou L Sagayaraj A Jumat NH Choolani M Chan JK. Hepatic differentiation of human amniotic epithelial cells and in *vivo* therapeutic effect on animal model of cirrhosis. J Gastroenterol Hepatol. (2015) 30:1673–82. doi: 10.1111/jgh.12991 25973537

[123] Zhang NN HG Han QB Hu X Yi J Yao L. Functional regeneration of irradiated salivary glands:with human amniotic epithelial cells transplantation. Int J Clin Exp Pathol. (2013) 6:2039–47. PMC3796225 24133581

[124] Yuge I Takumi Y Koyabu K Hashimoto S Takashima S Fukuyama T. Transplanted human amniotic epithelial cells express connexin 26 and Na-K-adenosine triphosphatase in the inner ear. Transplantation. (2004) 77:1452–4. doi: 10.1097/00007890-200405150-00023 15167605

[125] Wu Q Fang T Lang H Chen M Shi P Pang X. Comparison of the proliferation, migration and angiogenic properties of human amniotic epithelial and mesenchymal stem cells and their effects on endothelial cells. Int J Mol Med. (2017) 39:918–26. doi: 10.3892/ijmm.2017.2897 PMC5360425 28259958

[126] Liu QW Liu QY Li JY Wei L Ren KK Zhang XC. Therapeutic efficiency of human amniotic epithelial stem cell-derived functional hepatocyte-like cells in mice with acute hepatic failure. Stem Cell Res Ther. (2018) 9:321. doi: 10.1186/s13287-018-1063-2 30463600 PMC6249765

[127] Garcia-Castro IL Garcia-Lopez G Avila-Gonzalez D Flores-Herrera H Molina-Hernandez A Portillo W. Markers of pluripotency in human amniotic epithelial cells and their differentiation to progenitor of cortical neurons. PloS One. (2015) 10:e0146082. doi: 10.1371/journal.pone.0146082 26720151 PMC4697857

[128] Pratama G Vaghjiani V Tee JY Liu YH Chan J Tan C. Changes in culture expanded human amniotic epithelial cells: implications for potential therapeutic applications. PloS One. (2011) 6:e26136. doi: 10.1371/journal.pone.0026136 22073147 PMC3206797

[129] Evron A Goldman S Shalev E. Human amniotic epithelial cells cultured in substitute serum medium maintain their stem cell characteristics for up to four passages. Int J Stem Cells. (2011) 4:123–32. doi: 10.15283/ijsc.2011.4.2.123 PMC3840962 24298345

[130] Banas RA Trumpower C Bentlejewski C Marshall V Sing G Zeevi A. Immunogenicity and immunomodulatory effects of amnion-derived multipotent progenitor cells. Hum Immunol. (2008) 69:321–8. doi: 10.1016/j.humimm.2008.04.007 18571002

[131] Wu X Gao F Wu Y Sun R Guan W Tian X. Isolation and biological characteristics of sheep amniotic epithelial cells. Cytotechnology. (2019) 71:539–51. doi: 10.1007/s10616-019-00299-1 PMC6465395 30815768

[132] Diaz-Prado S Muinos-Lopez E Hermida-Gomez T Rendal-Vazquez ME Fuentes-Boquete I de Toro FJ. Multilineage differentiation potential of cells isolated from the human amniotic membrane. J Cell Biochem. (2010) 111:846–57. doi: 10.1002/jcb.22769 20665539

[133] Sugiura R Ohnishi S Ohara M Ishikawa M Miyamoto S Onishi R. Effects of human amnion-derived mesenchymal stem cells and conditioned medium in rats with sclerosing cholangitis. Am J Transl Res. (2018) 10:2102–14. PMC6079143 30093947

[134] Liu QW Li JY Zhang XC Liu Y Liu QY Xiao L. Human amniotic mesenchymal stem cells inhibit hepatocellular carcinoma in tumour-bearing mice. J Cell Mol Med. (2020) 24:10525–41. doi: 10.1111/jcmm.15668 PMC7521292 32798252

[135] Li JY Ren KK Zhang WJ Xiao L Wu HY Liu QY. Human amniotic mesenchymal stem cells and their paracrine factors promote wound healing by inhibiting heat stress-induced skin cell apoptosis and enhancing their proliferation through activating PI3K/AKT signaling pathway. Stem Cell Res Ther. (2019) 10:247. doi: 10.1186/s13287-019-1366-y 31399039 PMC6688220

[136] Bacenkova D Rosocha J Tothova T Rosocha L Sarissky M. Isolation and basic characterization of human term amnion and chorion mesenchymal stromal cells. Cytotherapy. (2011) 13:1047–56. doi: 10.3109/14653249.2011.592522 21916779

[137] Mihu CM Rus Ciuca D Soritau O Susman S Mihu D. Isolation and characterization of mesenchymal stem cells from the amniotic membrane. Rom J Morphol Embryol. (2009) 50:73–7. 19221648

[138] Nogami M Tsuno H Koike C Okabe M Yoshida T Seki S. Isolation and characterization of human amniotic mesenchymal stem cells and their chondrogenic differentiation. Transplantation. (2012) 93:1221–8. doi: 10.1097/TP.0b013e3182529b76 23318305

